# Eating disorders in times of the COVID‐19 pandemic—Results from an online survey of patients with anorexia nervosa

**DOI:** 10.1002/eat.23374

**Published:** 2020-08-25

**Authors:** Sandra Schlegl, Julia Maier, Adrian Meule, Ulrich Voderholzer

**Affiliations:** ^1^ Department of Psychiatry and Psychotherapy University Hospital, Ludwig‐Maximilians‐University Munich Germany; ^2^ Schoen Clinic Roseneck Prien am Chiemsee Germany; ^3^ Department of Psychiatry and Psychotherapy University Hospital of Freiburg Germany

**Keywords:** anorexia nervosa, coping strategies, COVID‐19 pandemic, health care utilization, symptoms worsening

## Abstract

**Objective:**

The COVID‐19 pandemic and the resulting public restrictions pose a psychological burden for humans worldwide and may be particularly detrimental for individuals with mental disorders. Therefore, the current study explored effects of the COVID‐19 pandemic on eating disorder (ED) symptoms and other psychological aspects in former inpatients with anorexia nervosa (AN).

**Method:**

One‐hundred and fifty‐nine patients with AN—discharged from inpatient treatment in 2019—completed an online survey on contact history with COVID‐19, changes in ED symptoms and other psychological aspects, health care utilization, and strategies patients employed to cope during the pandemic.

**Results:**

Approximately 70% of patients reported that eating, shape and weight concerns, drive for physical activity, loneliness, sadness, and inner restlessness increased during the pandemic. Access to in‐person psychotherapies and visits at the general practitioner (including weight checks) decreased by 37% and 46%, respectively. Videoconference therapy was used by 26% and telephone contacts by 35% of patients. Patients experienced daily routines, day planning and enjoyable activities as the most helpful among the most used coping strategies.

**Discussion:**

The COVID‐19 pandemic poses great challenges to patients with AN. ED‐related thoughts and behaviors may be used as dysfunctional coping mechanisms to regain control over the current circumstances. E‐mental health interventions appear to be promising for supporting AN patients during these hard times. Furthermore, interventions addressing symptoms of depression and anxiety, as well as intolerance of uncertainty might help them manage their ED symptoms.

## 1 INTRODUCTION

On 11th of March 2020, the World Health Organization declared the coronavirus disease 2019 (COVID‐19) a global pandemic. Governments imposed restrictions to slow down the spread of the disease to relieve the health system. Rules like social distancing and the shutdown of the societal life including schools and universities forced us to restructure our everyday life and social habits. An increase in the prevalence of anxiety and depression from 4% in 2019 to 20% in 2020 has been observed in the Chinese general population (Li et al., 2020). This raises concerns about the effects that the pandemic might have on existing mental illnesses.

Anorexia nervosa (AN) is a serious psychiatric illness that is associated with a low level of openness to unexpected events and avoidance of uncertainty (Hempel, Vanderbleek, & Lynch, 2018). Thus, weight, shape, and eating concerns may increase during times when the sense of self‐control decreases by external factors such as occurring in the present crisis (Fairburn, Shafran, & Cooper, 1999). An increase in eating disorder (ED) concerns may be further promoted by increased time spent on social media (Holland & Tiggemann, 2016). In addition, restrictions on doing grocery shopping may have multiple effects on eating behavior. On the one hand, they may foster skipping meals and restricting calories but, on the other hand, they may also increase binge eating due to the increased availability of food at home brought about by food insecurity and hoarding of food (Touyz, Lacey, & Hay, 2020; Weissman, Bauer, & Thomas, 2020). Thus, in times of uncertainty and instability, ED symptoms may increase as their functions can be providing control and/or safety.

However, due to the recency of the COVID‐19 pandemic, hardly any scientific studies are available. In a telephone survey of 32 patients with different EDs, 38% of patients reported increased ED symptoms and 56% reported an increase in anxiety (Fernandez‐Aranda et al., 2020). Most of the patients also expressed worries about adverse effects on their treatment (Fernandez‐Aranda et al., 2020). To ensure access to evidence‐based treatments, a reconsideration of standard treatment approaches (Touyz et al., 2020), an increased use of self‐help resources and e‐health interventions have been suggested (Weissman et al., 2020). Additionally, strategies and recommendations for dealing with the COVID‐19 pandemic have been proposed for patients with EDs as well as for their carers and therapists (Fernandez‐Aranda et al., 2020).

Due to the lack of empirical data on the impact of the current pandemic on patients with AN, the aim of this study was to explore psychological consequences of the COVID‐19 pandemic, changes in health care utilization, and possible strategies to cope with these times. Furthermore, differences in responses between adolescents and adults as well as between underweight and normal weight patients were analyzed exploratorily.

## 2 METHODS

### 2.1 Study participants

Former inpatients with AN who had received treatment at Schoen Clinic Roseneck (Prien am Chiemsee, Germany) and who were discharged in 2019 were contacted by e‐mail and invited to complete an anonymous online survey (duration approximately 15 minutes) via www.unipark.com. Inclusion criteria were (a) a primary diagnosis of AN (ICD‐10: F50.0 or F50.1) at admission, (b) female sex, and (c) age from 13 years onward.

### 2.2 Survey procedures and questions

Germany was in lockdown approximately from end of March to end of April 2020 (depending on the region), and the survey was conducted in the first week of May 2020. One reminder was sent 1 week later. The patients were told the following purpose of the study: “The COVID‐19 pandemic has profoundly changed our everyday lives. The associated challenges can influence the symptoms of mental illnesses. To systematically investigate its impact on patients with EDs, we would kindly ask you to take part in our survey.” Institutional review board approval and electronic informed patient consent were obtained.

We used a self‐developed (by a team of clinicians, psychotherapists and researchers) questionnaire (see Data S1) to assess psychological consequences of the COVID‐19 pandemic, which was divided into: (a) Sociodemographic and other information such as age, current self‐reported height and weight, occupational situation during the COVID‐19 pandemic, and contact history with SARS‐CoV‐2 (severe acute respiratory syndrome coronavirus 2); (b) Overall impact of the COVID‐19 pandemic on ED symptoms, incidence of new symptoms, quality of life, and therapy (5‐point scale with 1 = *strongly agree* to 5 = *strongly disagree*); (c) Changes during the pandemic regarding specific ED symptoms, exercise, and other eating‐related behaviors (5‐point scale from 1 = *significantly worsened/much more often* to 5 = *significantly improved/much less often*); (d) Changes during the pandemic regarding general psychopathology (depressive symptoms, anxiety, other general psychopathology symptoms) (5‐point scale with 1 = *significantly worsened* to 5 = *significantly improved*); (e) Worries, for example regarding infections, relapses, food insecurity, finances, and job (5‐point scale with 1 = *extremely worried* to 5 = *not at all worried*); (f) Changes during the pandemic regarding interpersonal conflicts (5‐point scale with 1 = *significantly worsened* to 5 = *significantly improved*); (g) Health care utilization before and during the COVID‐19 pandemic; (h) Use and helpfulness of coping strategies as suggested by Fernandez‐Aranda et al. (2020) (combination of yes/no answers and a 5‐point scale with 1 = *not helpful at all* to 5 = *very helpful*). All previous questions were mandatory and there was one optional open question: (i) “If you like, you can tell us here about your personal experiences or helpful strategies during the COVID‐19 pandemic. Also, if the pandemic has had any positive impact on your life or on your symptoms, you can report it here.”

### 2.3 Statistical analyses

Descriptive statistics of responses to all questions are presented. For questions that had a 5‐point scale response format, we combined the two categories of *somewhat agree/strongly agree*, *somewhat worried/extremely worried*, *somewhat worsened/significantly worsened*, and *more often/much more often* to explore whether responses would differ as a function of age (adolescents [age < 18 years] vs. adults [age ≥ 18 years]) and of body weight (underweight [BMI < 18.5 kg/m^2^] vs. normal weight [BMI ≥ 18.5 kg/m^2^]). Due to the exceedingly large number of comparisons, we present only effect sizes rather than *p*‐values. Cohen's *d* (0.2 = small effect, 0.5 = medium effect, and 0.8 = large effect) (Cohen, 1988) and number needed to take (NNT, 9 = small effect, 4 = medium effect, and 2 = large effect) (Kraemer, 2015; Kraemer, Neri, & Spiegel, 2020) were calculated to estimate effect sizes for continuous and categorical variables, respectively. NNT was initially developed to compare treatment outcomes (number of patients needed to be treated to prevent one “failure” or negative outcome that would have occurred had the patient received the control treatment). We applied NNT to address in our study the question of “how many adults do you have to ask to find one more “failure” (e.g., one more adult patient who endorses that the pandemic worsened their ED symptoms) than if you had sampled adolescents”? All analyses were conducted with IBM SPSS Statistics, version 24. Two authors (SS, JM) categorized all answers from the open‐ended question into broad themes. Any disagreement was resolved by consensus. We refrained from establishing a formal coding scheme since our goal was only to provide descriptive results. Example quotes for each theme are presented. Please note that the original quotes were translated from German into English.

## 3 RESULTS

### 3.1 Sample description

In total, 356 former inpatients with AN were contacted (90 adolescents and 266 adults). Of those, 241 patients accessed the survey. Thirty‐one patients only viewed the first page of the survey and 210 patients gave electronic informed consent. The final sample consisted of the 159 patients who completed the online survey. Thus, the overall participation rate was 59.0% (210/356) and the overall completion rate was 44.7% (159/356). The completion rate for adolescents was 52.2% (47/90) and for adults 42.1% (112/266). Table 1 shows the sample description of the entire sample and of the subgroups.

**TABLE 1:** Sociodemographic and clinical characteristics of former inpatients with anorexia nervosa

	Total sample (*N* = 159)	Adults (*n* = 112)	Adolescents (*n* = 47)	BMI < 18.5 (*n* = 92)	BMI ≥ 18.5 (*n* = 61)
**Age (years)**					
*M* (*SD*)	22.42 (8.67)	25.00 (9.16)	16.26 (0.92)	22.70 (9.25)	22.28 (8.17)
Range	14–62	18–62	14–17	14–62	14–52
Adults: n (%)	112 (70.4)	112 (100)	0 (0)	63 (68.5)	45 (73.8)
Adolescents: n (%)	47 (29.6)	0 (0)	47 (100)	29 (31.5)	16 (26.2)
**Body mass index (kg/m** ^**2**^ **)**					
*M* (*SD*) Range BMI < 18.5: *n* (%)	17.83 (2.43) 12.03–24.17 92 (60.1)	17.67 (2.55) 12.03–24.17 63 (56.3)	18.22 (2.09) 14.53–23.61 29 (61.7)	16.35 (1.66) 12.03–18.40 92 (100)	20.08 (1.50) 18.54–24.17 0 (0)
BMI ≥ 18.5: *n* (%)	61 (38.4)	45 (40.2)	16 (34.0)	0 (0)	61 (100)
**Occupational situation during the COVID‐19 pandemic: *n* (%)**					
Homeschooling	57 (35.8)	16 (14.3)	41 (87.2)	34 (37.0)	21 (34.4)
University online classes	32 (20.1)	32 (28.6)	0 (0)	19 (20.7)	11 (18.0)
Working from home	8 (5.0)	7 (6.3)	1 (2.1)	5 (5.4)	2 (3.3)
Working at workplace	29 (18.2)	27 (24.1)	2 (4.3)	14 (15.2)	14 (23.0)
Reduced working hours due to the pandemic	7 (4.4)	7 (6.3)	0 (0)	3 (3.3)	4 (6.6)
Job loss due to the pandemic	12 (7.5)	9 (8.0)	3 (6.4)	6 (6.5)	6 (9.8)
Other	14 (8.8)	14 (12.5)	0 (0)	11 (12.0)	3 (4.9)
**Infection with SARS‐CoV‐2: *n* (%)**					
Own infection	3 (1.9)	3 (2.7)	0 (0)	3 (3.3)	0 (0)
Infection in the household	3 (1.9)	2 (1.8)	1 (2.1)	3 (3.3)	0 (0)
Infection among related parties	11 (6.9)	8 (7.1)	3 (6.4)	7 (7.6)	3 (4.9)

*Notes:* BMI: missing data of *n* = 6 patients, so Ns and percentages do not total 159 and 100% respectively.

Abbreviations: BMI, body mass index; *M*, mean; SARS‐CoV‐2, severe acute respiratory syndrome coronavirus 2; *M*, mean; *SD*, standard deviation.

### 3.2 Impact of the COVID‐19 pandemic on symptoms in patients with AN

Table 2 shows the impact of the COVID‐19 pandemic on ED symptoms and other psychological aspects in patients with AN.

**TABLE 2:** Impact of the COVID‐19 pandemic on patients with anorexia nervosa

Overall impact					
	Strongly agree %	Agree %	Undecided %	Disagree %	Strongly disagree %
Worsening of eating disorder symptomatology	20.1	21.4	25.2	16.4	17.0
New symptoms	7.5	12.6	14.5	25.2	40.3
Worsening of quality of life	20.1	31.4	21.4	15.1	11.9
Impairment of therapy	13.2	14.5	20.8	28.9	22.6

Eating disorder symptoms, exercise, and other eating‐related behaviors					
	Much more %	More %	About the same %	Less %	Much less %
*Eating disorder cognitions*					
Drive for thinness	28.3	35.2	27.7	5.7	3.1
Fear of gaining weight	36.5	34.0	18.2	7.5	3.8
Body dissatisfaction	35.2	30.8	25.8	4.4	3.8
Eating concerns	42.1	32.7	18.2	3.1	3.8
Shape concerns	34.0	37.1	21.4	5.0	2.5
Weight concerns	29.6	37.1	25.2	5.0	3.1
Drive for physical activity	42.1	32.1	17.0	5.7	3.1
Eating disorder gives me control	28.3	27.0	35.2	3.1	6.3
Eating disorder gives me safety	30.2	29.6	31.4	1.9	6.9
*Eating disorder behaviors*					
Restrictive eating	13.8	33.3	38.4	7.5	6.9
Binge‐eating (>1,000 kcal)	4.4	10.1	78.0	1.3	6.3
Self‐induced vomiting	6.3	5.7	82.4	0	5.7
Laxative abuse	1.9	5.0	86.2	1.3	5.7
Diuretic abuse	1.3	1.3	91.2	0.6	5.7
Weighing oneself	11.9	18.2	59.1	5.7	5.0
Hoarding food	3.1	20.1	69.8	3.8	3.1
Snacking/unplanned eating	8.8	15.7	64.2	3.1	8.2
Skip meals	11.3	23.9	50.3	6.9	7.5
Sleep in and skip breakfast	8.8	13.8	62.9	4.4	10.1
Consuming triggering social media	13.8	27.0	52.8	2.5	3.8
*Exercise behaviors*					
Going for a walk	32.1	40.3	17.6	6.9	3.1
Jogging	17.0	20.8	55.3	1.3	5.7
Home‐workouts	25.8	37.7	32.1	2.5	1.9
Standing on purpose	9.4	21.4	60.4	3.1	5.7
Taking stairs	8.8	20.1	64.8	5.0	1.3
*Other eating‐related behaviors*					
Daily routine	6.3	15.1	33.3	32.7	12.6
Grocery shopping	7.5	16.4	23.9	29.6	22.6
Time for meal preparation	13.2	35.2	42.1	6.3	3.1
Going out for dinner	0	2.5	15.1	11.3	71.1
Cooking	19.5	29.6	35.2	8.2	7.5
Regular meal structure	8.8	20.1	45.9	14.5	10.7
Eating alone	17.0	19.5	34.0	14.5	15.1

General psychopathology symptoms					
	Significantly worsened %	Somewhat worsened %	Neither nor %	Somewhat improved %	Significantly improved %
*Depressive symptoms*					
Sadness	30.8	40.9	21.4	3.8	3.1
Loss of pleasure	30.2	35.2	30.2	1.3	3.1
Loss of interest	22.6	32.1	38.4	3.8	3.1
Loss of energy	28.9	30.2	30.2	8.2	2.5
Loneliness	41.5	35.2	17.0	5.0	1.3
Sleep disturbances	21.4	37.1	34.6	5.0	1.9
Hypersomnia	9.4	33.3	54.7	2.5	0
Changes in appetite	11.9	30.8	49.7	4.4	3.1
Worthlessness	23.3	34.0	38.4	2.5	1.9
Suicidal thoughts	10.1	23.9	60.4	1.3	4.4
*Anxieties*					
Fear something bad may happen	11.3	27.0	57.9	3.1	0.6
Fear not being able to stop or control worries	17.6	35.2	45.3	1.3	0.6
Fear of contact with others	11.9	25.8	56.6	3.1	2.5
Worries that feelings get out of control	23.3	28.3	45.3	1.9	1.3
*Other general psychopathology symptoms*					
Motor restlessness	23.9	38.4	33.3	3.1	1.3
Inner restlessness	30.8	41.5	22.6	3.1	1.9
Loss of control	34.0	29.6	31.4	2.5	2.5
Concentration difficulty	17.0	31.4	45.3	5.0	1.3
Self‐harm	6.3	14.5	71.7	4.4	3.1
Alcohol	2.5	6.9	84.3	2.5	3.8

Worries					
	Extremely worried %	Moderately worried %	Undecided %	Slightly worried %	Not at all worried %
Own infection	6.3	11.9	13.2	36.5	32.1
Infection of others (e.g. family or friends)	18.9	34.0	21.4	16.4	9.4
To infect others	16.4	23.3	17.6	19.5	23.3
Negative impact on therapy	15.1	13.2	23.9	20.1	27.7
Relapse	36.5	14.5	20.8	12.6	15.7
Food insecurity (i.e., availability, access)	8.8	9.4	11.9	18.9	50.9
Financial situation	8.8	11.9	10.1	25.8	43.4
Loss of job	4.4	6.3	3.8	14.5	71.1

Interpersonal conflicts					
	Significantly worsened %	Somewhat worsened %	Neither nor %	Somewhat improved %	Significantly improved %
Relationships conflicts	4.4	8.8	84.3	1.3	1.3
Friendship conflicts	1.3	13.2	78.0	4.4	3.1
Family conflicts	17.0	29.6	39.6	10.1	3.8
Conflicts in the workplace	0.6	5.0	89.3	2.5	2.5

#### 3.2.1 COVID‐19‐related impact on ED symptoms, exercise, and other eating‐related behaviors

Regarding the question whether the pandemic worsened their ED symptoms, one in four patients indicated that they were “undecided”; of those reporting a change, more patients agreed or strongly agreed (41.5%) than disagreed or strongly disagreed (33.4%) that their symptoms had gotten worse. New symptoms were reported by about 20% of patients, while 65.5% negated experiencing new symptoms. Regarding specific symptoms, more than 70% of patients reported that ED cognitions such as eating and shape concerns, drive for physical activity, and fear of gaining weight increased. In more than 60% of patients, going for a walk and at‐home workouts increased. However, more than 50% of patients indicated that ED behaviors such as restrictive eating, skipping meals, binge eating and purging were unchanged or in some cases even less or much less frequent; half of patients had more time for meal preparation and cooked more. Moreover, 73% of patients also reported that they maintained their weight (± 2 kg), whereas 18.9% reported a weight decrease (*M* = 4.25 kg, *SD* = 1.72, range: 2.5–10 kg) and 8.2% reported a weight increase (*M* = 4.17 kg, *SD* = 2.75, range: 2.5–11 kg).

#### 3.2.2 COVID‐19‐related impact on depression and anxiety symptoms, other general psychopathology symptoms, worries and interpersonal conflicts

Slightly over half of patients reported a deterioration of their quality of life while only 17% indicated no worsening of it. More than 70% of the sample reported that loneliness, inner restlessness, and sadness increased. Half of patients indicated fears of not being able to stop or control worries and worries that feelings get out of control. Most reported worries were related to infection of others and relapse (each about 50%), but more than two thirds of patients were not worried about an own infection, their financial situation, food insecurity and/or job loss. 46.6% reported increases in family conflicts, whereas more than 80% reported no changes regarding friendship and relationship conflicts and/or conflicts in the workplace.

### 3.3 Impact of the COVID‐19 pandemic on health care utilization

The percentage of patients receiving in‐person outpatient psychotherapy decreased from 88.1% to 55.3%, weighing by a clinician or a therapist from 48.4% to 30.8% and visits at the general practitioner from 44.0% to 23.9%. In contrast, phone contacts with therapists increased from 12.6% to 35.2%, videoconference therapy from 1.3% to 25.8% and additional online interventions from 3.1% to 6.9%. 8.2% of patients were in inpatient treatment during the pandemic, at least part of the time. 10.7% of patients that had been receiving therapy before the pandemic did not get any therapy during the pandemic.

### 3.4 Use and helpfulness of coping strategies during the COVID‐19 pandemic

**FIGURE 1:**
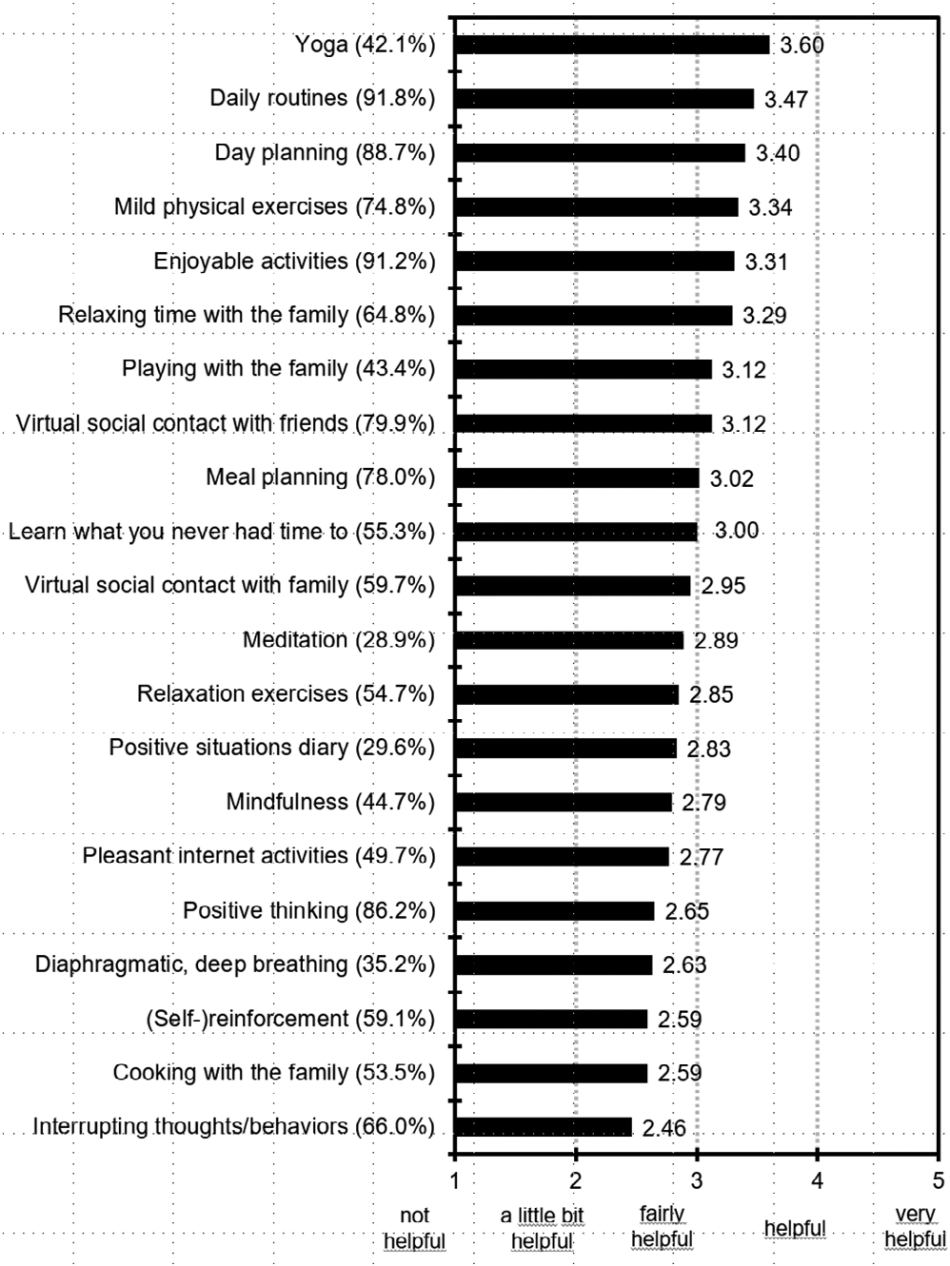
Mean responses to questions on helpfulness of coping strategies. Higher scores represent higher helpfulness ratings. The values in parentheses reflect the percentages of patients that used the strategies

The strategies rated most useful among those most used by patients with AN were daily routines, day planning, enjoyable activities and mild physical activities. Figure 1 shows the mean responses to questions on the use and helpfulness of different strategies.

### 3.5 Positive consequences of the COVID‐19 pandemic

Forty‐one (25.8%) of patients responded to the open‐ended question. We only extracted answers related to positive consequences since most answers were related to this aspect. We identified “reduction in overall ED symptoms/taking on responsibility to recover”, “reduction in specific ED symptoms”, “more flexibility regarding meals and foods”, “wake‐up call/will to live”, “trying out therapy content” and “accepting uncertainty in life” as broad themes. Exemplary quotes of the broad themes are given in Table 3.

**TABLE 3:** Positive consequences of the COVID‐19 pandemic reported by patients with anorexia nervosa

Theme	Example quote
Reduction in overall eating disorder symptoms/Taking on responsibility to recover	“Funnily enough, because I can't expect much help from outside at the moment, I got my ED under control quite well.”
Reduction in specific eating disorder symptoms	“I voluntarily went into quarantine at home for 2 weeks to stop my excessive exercise behavior.”
More flexibility regarding meals and foods	“New resolutions: Snacking twice a week late in the evening, eating difficult foods four times a week, always trying my friend's food if he offers it to me.”
“Wake‐up call”/Will to live	“The threat from the virus woke me up. Because anorexia and pneumonia just do not get along so well. For me personally, this pandemic opened my eyes in a way and showed me that I want to live.”
Trying out therapy content	“A lot of time to rethink, rework and try out content of therapy sessions.”
Accepting uncertainty in life	“I understand even better that life cannot be planned. At the beginning of the pandemic, I was very concerned about what to do with all the free time, but it was incredibly good for me to learn how to deal with this uncertainty, not knowing what the new day will bring…I was able to learn to pay attention to my feelings, to accept that there are good and bad days and, above all, to learn to simply live in the day and spontaneously decide what I want and how I want to organize this day.”

### 3.6 Responses as a function of age and body weight

In Data S2, responses of patients with AN are presented for adults vs. adolescents and underweight vs. normal weight patients, respectively.

When comparing adults and adolescents with AN (see Tables S1 and S2), we found medium effects with adults reporting more worries that others might be infected and with adolescents of being more frequently weighed by a therapist or a general practitioner before and during the pandemic. Adolescents also rated mindfulness and the opportunity to learn what one never had time to do as more helpful. Additionally, there were several small effects with adults being in general slightly more affected and reporting a somewhat greater impairment of therapy than adolescents, with the exception that the latter ones reported somewhat more positive eating‐related behaviors. Drive for physical activity and exercise behaviors were relatively comparable between the two groups.

When comparing underweight and normal weight patients with AN (see Tables S3 and S4), we found medium effects for underweight patients who rated relaxation exercises and meditation as more helpful than normal weight patients. We found no differences regarding depressive symptoms and worries, but several small effects regarding other symptoms and health care utilization.

## 4 DISCUSSION

This is the first survey that comprehensively investigated the impact of the COVID‐19 pandemic in a large sample of former inpatients with AN.

### 4.1 Summary of results

While overall more patients with AN reported a worsening of symptoms, a considerable subset disagreed that their symptoms had worsened. An emergence of new symptoms was the exception and most patients succeeded in maintaining their weight. Quality of life deteriorated in half of patients, and many patients reported increases in depression and anxiety symptoms. Despite a decreased access to in‐person psychotherapy and visits at the general practitioner including weight checks, only one in five patients reported that their treatment was deeply impaired by the pandemic. Finally, most of the patients used several coping strategies and experienced these as fairly helpful to helpful.

### 4.2 Impact on ED symptoms

A substantial subset of patients reported an increase in ED cognitions and drive for physical activity. Although our data are cross‐sectional and preclude discussion of temporal or causal relationships, we hypothesize that increased symptoms of depression and anxiety experienced during the pandemic may have contributed to an increased drive for physical activity as one of the functions exercising serves in patients with AN is affect regulation (Meyer, Taranis, Goodwin, & Haycraft, 2011). Similarly, focusing attention on weight and shape might have been a maladaptive strategy to cope with intolerance of uncertainty, and symptoms of depression and anxiety. In line with this, a study by Frank et al. (2012) found that intolerance of uncertainty was positively correlated with depression, drive for thinness and body dissatisfaction. Furthermore, a study by Naumann, Tuschen‐Caffier, Voderholzer, and Svaldi (2014) reported that rumination in response to sadness (which was the most prevalent depressive symptom in our sample) had detrimental effects on ED symptoms.

Although a substantial subset of patients reported an overall worsening of their ED symptoms, many reported that the pandemic did not or only slightly affect their ED symptoms and some even reported improvement of symptoms. Furthermore, most patients succeeded in maintaining their weight. There are several potential reasons to explain this finding. First, all patients were former inpatients and, thus, they might have used some of the adaptive coping strategies they acquired during treatment. Second, most patients indicated that they were still receiving therapy during the pandemic, which likely helped to prevent relapse. Third, patients had a relatively high BMI and might have already experienced self‐efficacy regarding managing their ED. Finally, there might have been at least a subset of patients (especially the younger ones) who returned to their parents' place and might have had a more regular meal structure, and experienced more social connectedness (despite the potential of increased family conflicts).

### 4.3 Impact on symptoms of depression and anxiety

Many patients also indicated that symptoms of depression and anxiety increased during the COVID‐19 pandemic. These results are in line with previous studies that have shown increases in symptoms of depression and anxiety, and loneliness in the general population (Fitzpatrick, Harris, & Drawve, 2020; Li et al., 2020). Furthermore, we investigated a sample of women, probably mostly unmarried, two factors that contributed to a higher likelihood of experiencing depressive symptoms during the COVID‐19 pandemic in the general population (Fitzpatrick et al., 2020). Moreover, intolerance of uncertainty has been found to be a risk factor for symptoms of depression and anxiety in general (Mahoney & McEvoy, 2012). As intolerance of uncertainty is highly prevalent in individuals with EDs (Brown et al., 2017; Kesby, Maguire, Brownlow, & Grisham, 2017), feelings of uncertainty due to the COVID‐19 pandemic may have contributed to increases in symptoms of depression and anxiety in the current sample. Also, the majority of patients reported increases in loneliness during the pandemic, which has been identified as a predictor of depressive symptoms in general (Cacioppo, Hughes, Waite, Hawkley, & Thisted, 2006). Accordingly, more than half of the lonely participants met criteria for depression during the COVID‐19 pandemic in the general population (Killgore, Cloonan, Taylor, & Dailey, 2020).

### 4.4 Impact on health care utilization

Regarding health services use during the pandemic, we found a decreased access to in‐person psychotherapy, but only a third of patients with AN used videoconference therapy and/or online interventions. This is not in line with suggestions that the COVID‐19 pandemic might be a turning point for e‐health (Torous & Wykes, 2020; Wind, Rijkeboer, Andersson, & Riper, 2020). Also, the editorials on the COVID‐19 pandemic and EDs suggested to use digital interventions to ensure access to evidence‐based treatments (Fernandez‐Aranda et al., 2020; Touyz et al., 2020; Weissman et al., 2020), and guidance for delivering distance cognitive‐behavioral therapy and family‐based treatment for EDs is available (Matheson, Bohon, & Lock, 2020; Murphy, Calugi, Cooper, & Dalle Grave, 2020; Waller et al., 2020). For a general discussion on the use of digital technology in the treatment of EDs also refer to Taylor, Fitzsimmons‐Craft, and Graham (2020). Our findings suggest that e‐mental‐health interventions are still not widely used, at least in Germany. This is somewhat surprising as a recent survey of attitudes toward e‐therapy interventions for ED psychopathology found positive attitudes towards ED‐related e‐mental‐health interventions in a sample from the general population (Linardon, Shatte, Tepper, & Fuller‐Tyszkiewicz, 2020), especially in those with bulimic symptoms. However, patients with AN also expressed ambivalent feelings towards video calls (Fernandez‐Aranda et al., 2020) suggesting that the rare use of e‐health interventions in the current sample may be partially explained by the unwillingness of the patients to participate in such interventions. On the other hand, therapists may also have hesitated to use new technologies in treatment during the pandemic.

### 4.5 Use and helpfulness of coping strategies

As for useful coping strategies, daily routines, day planning, enjoyable activities and mild physical activities were experienced as most useful by patients with AN. This is in line with suggestions for achieving good mental health during COVID‐19 social isolation by Diamond and Willan (2020) who recommended among other things that building a routine, physical activity (even light physical activity) and mindfulness might be beneficial.

### 4.6 Responses as a function of age and body weight

Regarding exploratory subgroup analyses, our findings suggest a somewhat different impact of the pandemic as a function of age and body weight with adults and underweight patients being slightly more affected than adolescents and normal weight patients. To the best of our knowledge, no studies exist so far that compare mental health consequences of the pandemic between adults and adolescents, neither in the general population nor in patients with mental health conditions. As differences between age and body weight groups were mostly of small magnitude, future evidence is warranted that corroborates these findings.

### 4.7 Clinical implications

Our findings may have some implications for treating patients with AN during the pandemic. For example, strategies for learning to control the ED mindset such as described by Fairburn (2008) might be useful. Given the increases in symptoms of depression and anxiety, interventions aimed at helping patients cope with these symptoms might help manage their ED symptoms. As Cooper et al. (2020) have suggested, behavioral activation (such as activity monitoring and scheduling or assessment of goals and values) might be indicated to treat increased depressive symptoms and also holds promise in treating anxiety (Stein, Carl, Cuijpers, Karyotaki, & Smits, 2020). In addition, elements from acceptance and commitment therapy (ACT) (Hayes, Strosahl, & Wilson, 1999) might be applied to help patients better cope with intolerance of uncertainty. ACT might also be used to help patients explore life goals and values and to motivate or support them to view the pandemic as an opportunity to rethink life.

### 4.8 Limitations

Several limitations should be considered when interpreting the current results. First, we only assessed women with AN who were discharged from inpatient treatment the year before and had a relatively high mean BMI. Therefore, results may not be transferable to women with AN who have not yet received therapy or are still at the very beginning and to other persons with other EDs. Second, all data were self‐reported and, thus, may potentially be biased. For example, it cannot be excluded that at least some symptom worsening was not directly attributable to the COVID‐19 pandemic. Furthermore, body weight was based on self‐report, yet, women with AN are very accurate when self‐reporting their own weight (McCabe, McFarlane, Polivy, & Olmsted, 2001; Meyer, Arcelus, & Wright, 2009). Third, we only assessed how often e‐mental‐health interventions were used, but not how often they were offered. Thus, it might be that the small percentage of patients using such interventions might not be attributable to a lack of interest or an unwillingness on patients' side, but to the fact that therapists did not offer such interventions.

### 4.9 Future research

Future studies should explore whether there are more hindering factors on therapists' or on patients' side for using e‐health interventions. Furthermore, exploring the view and burden of caregivers and/or therapists during the pandemic might be interesting. Finally, longitudinal analyses might help shed light on how long lasting the negative effects reported by the patients are and if they will remit spontaneously or if additional treatment is needed.

## 5 CONCLUSION

Taken together, this study suggests that during the COVID‐19 pandemic, patients with AN were at risk for deterioration of ED and general symptoms and help‐seeking may increase after the crisis. More flexible treatment modalities should be considered in treatment and relapse prevention.

## CONFLICT OF INTEREST

The authors declare no potential conflict of interest.

## Supporting information


**Appendix** **S1**: Supporting Information

**Appendix** **S2**: Supporting Information

## Data Availability

The data that support the findings of this study are available from the corresponding author upon reasonable request.
