# Supplementary material for: Eating disorders in times of the COVID‐19 pandemic—Results from an online survey of patients with anorexia nervosa
Source: Int J Eat Disord. 2020 Aug 25;53(11):1791–800. doi: 10.1002/eat.23374 (PMC7461418; doi:10.1002/eat.23374)
Supplement: Supplementary file 1 — Appendix S1: Supporting Information [file EAT-53-1791-s001.docx]

***Supplemental Material 1: Survey on the impact of the COVID-19 pandemic on patients with eating disorders***

**Sandra Schlegl, Julia Maier, Adrian Meule, Ulrich Voderholzer**

Note: the questionnaire was used in German and translated into English for the purpose of this supplemental material.

Dear patient,

the COVID-19 pandemic has profoundly changed our everyday lives and many areas of our lives. The associated challenges can influence the symptoms of mental illnesses. To systematically investigate its impact on patients with eating disorders, we would kindly ask you to take part in our survey.

In this questionnaire, we ask you for information about the possible effects of the COVID-19 pandemic in your life.

We ask you to answer all questions carefully and honestly. It is important that there are no answers that are right or wrong. We care about what you think and feel, not what seems right or wrong.

Participation in this study is voluntary. You can end your participation at any time and without giving reasons by closing the window browser. This will have no negative consequences for you.

All data is collected anonymously, i.e. you will never be asked to give your name or any other information identifying you.

Yes, I agree that my personal data will be processed according to the information given here.

No, I do not wish to participate.

**1. Sociodemographic and other information**

**Age (in years):** __________

**Size (cm)** _____

**Current weight (kg):** _____

**Eating disorder:**

1. Anorexia nervosa
2. Bulimia nervosa
3. Other:___________________

**Occupational situation during the COVID-19 pandemic:**

1. Homeschooling
2. University online classes
3. Working from home
4. Working at workplace
5. Reducing working hours due to the COVID-19 pandemic
6. Job loss due to the COVID-19 pandemic
7. Other:_____________________

**Infection with SARS-CoV-2 (severe acute respiratory syndrome coronavirus 2):**

Are you currently infected with SARS-CoV-2
(confirmed with a positive test result) Yes No

Have you ever been infected with SARS-CoV-2? Yes No

Are there any confirmed cases in your household? Yes No

Are there any confirmed cases among related parties? Yes No

**2. Overall impact of the COVID-19 pandemic**

| **General personal impact of the COVID-19 pandemic** | 1 2 3 4 5 Strongly undecided strongly agree disagree |
| --- | --- |
| 1. The COVID-19 pandemic worsened my eating disorder symptomatology. |  |
| 1. The COVID-19 pandemic has deteriorated my quality of life. |  |
| 1. The COVID-19 pandemic deeply impaired my therapy. |  |
| 1. During the COVID-19 pandemic new symptoms have developed. |  |
| If new symptoms have developed during the COVID-19 pandemic: which ones? ________________________________________________________________________________ ________________________________________________________________________________  ________________________________________________________________________________ | |

**3. Eating disorder symptoms and behaviors as well as other exercise and eating-related bevaviors**

| **How much have the following eating disorder symptoms changed during the COVID-19 pandemic?** | 1 2 3 4 5  Significantly neither/ significantly worsened/ nor improved/  increased decreased |
| --- | --- |
| 1. Drive for thinness |  |
| 1. Fear of gaining weight |  |
| 1. Body dissatisfaction |  |
| 1. Eating concerns |  |
| 1. Shape concerns |  |
| 1. Weight concerns |  |
| 1. Drive for physical activity |  |
| 1. Feeling that the eating disorder helps me to have everything under control |  |
| 1. Feeling that the eating disorder gives me safety |  |

| **How much have the following behaviors changed during the COVID 19 pandemic?** | 1 2 3 4 5  Much more neither/ much less often nor often |
| --- | --- |
| 1. Restrictive eating |  |
| 1. Binge-eating (>1000 kcal) |  |
| 1. Self-induced vomiting |  |
| 1. Laxative abuse |  |
| 1. Diuretic abuse |  |
| 1. Weighing oneself |  |
| 1. Hoarding food |  |
|  |  |
| 1. Snacking/unplanned eating |  |
| 1. Skip meals |  |
| 1. Sleep in and skip breakfast |  |
| 1. Consuming triggering social media |  |
| 1. Going for a walk |  |
| 1. Jogging |  |
| 1. Home-workouts |  |
| 1. Standing on purpose |  |
| 1. Taking stairs |  |
| 1. Daily routine |  |
| 1. Grocery shopping |  |
| 1. Time for meal preparation |  |
| 1. Going out for dinner |  |
| 1. Cooking |  |
| 1. Regular meal structure |  |
| 1. Eating alone |  |

**4. General psychopathology symptoms**

| **How much have the following general symptoms changed during the COVID-19 pandemic?** | 1 2 3 4 5  Significantly neither/ significantly worsened/ nor improved/  increased decreased |
| --- | --- |
| 1. Sadness |  |
| 1. Loss of pleasure |  |
| 1. Loss of interest |  |
| 1. Loss of energy |  |
| 1. Loneliness |  |
| 1. Sleep disturbances |  |
| 1. Hypersomnia |  |
| 1. Changes in appetite |  |
| 1. Worthlessness |  |
| 1. Suicidal thoughts |  |
| 1. Fear something bad may happen |  |
| 1. Fear not being able to stop or control worries |  |
| 1. Fear of contact with others |  |
| 1. Worries that feelings get out of control |  |
| 1. Motor restlessness |  |
| 1. Inner restlessness |  |
| 1. Loss of control |  |
| 1. Concentration difficulty |  |
| 1. Self-harm |  |
| 1. Alcohol consumption |  |
| 1. Relationship conflicts |  |
| 1. Friendship conflicts |  |
| 1. Family conflicts |  |
| 1. Conflicts in the workplace |  |

**5. Worries**

| **How much do/did you feel worried by the following worries related to the COVID-19 pandemic?** | 1 2 3 4 5  Extremely undecided not at all  worried worried |
| --- | --- |
| 1. Worries regarding an own infection |  |
| 1. Worries regarding infections of others |  |
| 1. Worries to infect others |  |
| 1. Worries regarding a negative impact on therapy |  |
| 1. Worries regarding relapse |  |
| 1. Worries regarding food insecurity (i.e. availability, access) |  |
| 1. Worries regarding financial situation |  |
| 1. Worries regarding loss of job |  |

**6. Interpersonal conflicts**

| **How much have the following general symptoms changed during the COVID-19 pandemic?** | 1 2 3 4 5  Significantly neither/ significantly worsened/ nor improved/  increased decreased |
| --- | --- |
| 1. Relationship conflicts |  |
| 1. Friendship conflicts |  |
| 1. Family conflicts |  |
| 1. Conflicts in the workplace |  |

**7. Health care utilization**

| **How often did you**  **use the following services?** | Before the COVID-19 pandemic | | During the COVID-19 pandemic |
| --- | --- | --- | --- |
| In-person psychotherapy | Never  1x /week  Several times a week  ever 2 weeks  1x /month | | Never  1x /week  Several times a week  ever 2 weeks  1x /month |
| Videoconference therapy | Never  1x /week  Several times a week  ever 2 weeks  1x /month | | Never  1x /week  Several times a week  ever 2 weeks  1x /month |
| Telephone contact | Never  1x /week  Several times a week  ever 2 weeks  1x /month | | Never  1x /week  Several times a week  ever 2 weeks  1x /month |
| General practitioner visit | Never  1x /week  Several times a week  ever 2 weeks  1x /month | | Never  1x /week  Several times a week  ever 2 weeks  1x /month |
| Weighing (by therapist or general practitioner) | Never  1x /week  Several times a week  ever 2 weeks  1x /month | | Never  1x /week  Several times a week  ever 2 weeks  1x /month |
| Add-on online intervention | Never  1x /week  Several times a week  ever 2 weeks  1x /month | | Never  1x /week  Several times a week  ever 2 weeks  1x /month |
| Have you been in inpatient treatment during COVID-19 pandemic | | Yes  No | |

**8. Coping strategies**

| **Which of the following strategies did you use during the COVID-19 pandemic and, if you used them, how helpful did you find these strategies?** | \| 0 \| 1 \| 2 \| 3 \| 4 \| 5 \| \| --- \| --- \| --- \| --- \| --- \| --- \| \| Not used \| Not helpful \|  \| neither/ nor \|  \| very helpful \| |
| --- | --- | --- | --- | --- | --- | --- | --- | --- | --- | --- | --- | --- | --- |
| 1. Strategies for interrupting thoughts and pathological behavior |  |
| 1. Positive thinking |  |
| 1. Day planning |  |
| 1. Daily routines |  |
| 1. Enjoyable activities |  |
| 1. Virtual social contact (friends) |  |
| 1. Virtual social contact (family) |  |
| 1. Mild physical exercises |  |
| 1. Relaxation exercises |  |
| 1. Meal planning |  |
| 1. Yoga |  |
| 1. Cooking with the family |  |
| 1. Diaphragmatic and deep breathing |  |
| 1. Meditation |  |
| 1. Mindfulness |  |
| 1. Seeking reinforcement and self-reinforcement |  |
| 1. Keeping a diary of positive situations |  |
| 1. Relaxing time with the family |  |
| 1. Playing with the family |  |
| 1. Opportunity to learn what you never had time to do |  |
| 1. Pleasant activities on the internet |  |

**9. Open Question:**

If you like, you can tell us here about your personal experiences or helpful strategies during the COVID-19 pandemic. Also, if the pandemic has had any positive impact on your life or on your symptoms, you can report it here.

__________________________________________________________________________

__________________________________________________________________________

__________________________________________________________________________

__________________________________________________________________________

__________________________________________________________________________

__________________________________________________________________________

**Thank you very much for your participation!**
