# Supplementary material for: Eating disorders in times of the COVID‐19 pandemic—Results from an online survey of patients with anorexia nervosa
Source: Int J Eat Disord. 2020 Aug 25;53(11):1791–800. doi: 10.1002/eat.23374 (PMC7461418; doi:10.1002/eat.23374)
Supplement: Supplementary file 2 — Appendix S2: Supporting Information [file EAT-53-1791-s002.docx]

**Supplemental Material 2: Subgroup analyses**

**1) Responses as a function of age**

Supplemental Table 1: Impact of the COVID-19 pandemic on patients with anorexia nervosa as a function of age

|  | Adults  % | Adolescents  % | NNT |
| --- | --- | --- | --- |
| **Overall impact** | | | |
| Worsening of eating disorder symptomatology | 42.0 | 40.4 | 63 |
| New symptoms | 21.4 | 17.0 | 23 |
| Worsening of quality of life | 51.8 | 51.1 | 143 |
| Impairment of therapy | 31.3 | 19.1 | 8 |
| **Eating disorder symptoms, exercise, and other eating-related behaviors** | | | |
| Eating disorder cognitions | | | |
| Drive for thinness | 67.9 | 53.2 | 7 |
| Fear of gaining weight | 71.4 | 68.1 | 30 |
| Body dissatisfaction | 71.4 | 53.2 | 5 |
| Eating concerns | 76.8 | 70.2 | 15 |
| Shape concerns | 73.2 | 66.0 | 14 |
| Weight concerns | 71.4 | 55.3 | 6 |
| Drive for physical activity | 75.0 | 72.3 | 37 |
| ED gives me control | 58.0 | 48.9 | 11 |
| ED gives me safety | 63.4 | 51.1 | 8 |
| Eating disorder behaviors | | | |
| Restrictive eating | 51.8 | 36.2 | 6 |
| Binge-eating (>1000 kcal) | 14.3 | 14.9 | -167 |
| Self-induced vomiting | 13.4 | 8.5 | 20 |
| Laxative abuse | 8.0 | 4.3 | 27 |
| Diuretic abuse | 3.6 | 0 | 28 |
| Weighing oneself | 33.0 | 23.4 | 10 |
| Hoarding food | 24.1 | 21.3 | 36 |
| Snacking/Unplanned eating | 25.9 | 21.3 | 22 |
| Skip meals | 36.6 | 31.9 | 21 |
| Sleep in and skip breakfast | 23.2 | 21.3 | 53 |
| Consuming triggering social media | 38.4 | 46.8 | -12 |
| Exercise behaviors | | | |
| Going for a walk | 71.4 | 74.5 | -32 |
| Jogging | 35.7 | 42.6 | -14 |
| Home-workouts | 62.5 | 66.0 | -29 |
| Standing on purpose | 28.6 | 36.2 | -13 |
| Taking stairs | 29.5 | 27.7 | 56 |
| Other eating-related behaviors | | | |
| Daily routine | 20.5 | 23.4 | -34 |
| Grocery shopping | 22.3 | 27.7 | -19 |
| Time for meal preparation | 42.9 | 61.7 | -5 |
| Going out for dinner | 1.8 | 4.3 | -40 |
| Cooking | 43.8 | 61.7 | -6 |
| Regular meal structure | 25.9 | 36.2 | -10 |
| Eating alone | 35.7 | 38.3 | -38 |
| **Weight change** | | | |
| Weight decrease | 24.1 | 6.4 | 5.65 |
| Weight maintenance | 67.9 | 85.1 | -5.81 |
| Weight increase | 8 | 8.5 | -200.00 |
| **General psychopathology symptoms** | | | |
| Depressive symptoms | | | |
| Sadness | 73.2 | 68.1 | 20 |
| Loss of pleasure | 66.1 | 63.8 | 43 |
| Loss of interest | 55.4 | 53.2 | 45 |
| Loss of energy | 58.9 | 59.6 | -143 |
| Loneliness | 79.5 | 70.2 | 11 |
| Sleep disturbances | 64.3 | 44.7 | 5 |
| Hypersomnia | 44.6 | 38.3 | 16 |
| Changes in appetite | 42.9 | 42.6 | 333 |
| Worthlessness | 61.6 | 46.8 | 7 |
| Suicidal thoughts | 33.0 | 36.2 | -31 |
| Anxieties | | | |
| Fear something bad may happen | 40.2 | 34.0 | 16 |
| Fear not being able to stop or control worries | 53.6 | 51.1 | 40 |
| Fear of contact with others | 33.9 | 46.8 | -8 |
| Worries that feelings get out of control | 49.1 | 57.4 | -12 |
| Other general psychopathology symptoms | | | |
| Motor restlessness | 66.1 | 53.2 | 8 |
| Inner restlessness | 75.9 | 63.8 | 8 |
| Loss of control | 64.3 | 61.7 | 38 |
| Concentration difficulty | 43.8 | 59.6 | -6 |
| Self-harm | 20.5 | 21.3 | -125 |
| Alcohol | 12.5 | 2.1 | 10 |
| **Worries** | | | |
| Own infection | 18.8 | 17.0 | 56 |
| Infection of others (e.g. family or friends) | 58.9 | 38.3 | 5 |
| To infect others | 47.3 | 21.3 | 4 |
| Negative impact on therapy | 30.4 | 23.4 | 14 |
| Relapse | 53.6 | 44.7 | 11 |
| Food insecurity (i.e. availability, access) | 17.9 | 19.1 | -83 |
| Financial situation | 26.8 | 6.4 | 5 |
| Loss of job | 13.4 | 4.3 | 11 |
| **Interpersonal conflicts** | | | |
| Relationships conflicts | 14.3 | 10.6 | 27 |
| Friendship conflicts | 13.4 | 17.0 | -28 |
| Family conflicts | 40.2 | 61.7 | -5 |
| Conflicts in the workplace | 6.3 | 4.3 | 50 |
| **Health care utilization before and during the COVID-19 pandemic** | | | |
| In-person psychotherapy before | 86.6 | 91.5 | -20 |
| In-person psychotherapy during | 55.4 | 55.3 | 1000 |
| Videoconference therapy before | 0.9 | 2.1 | -83 |
| Videoconference therapy during | 27.7 | 21.3 | 16 |
| Telephone contacts before | 15.2 | 6.4 | 11 |
| Telephone contacts during | 33.0 | 40.4 | -14 |
| GP visits before | 45.5 | 40.4 | 20 |
| GP visits during | 24.1 | 23.4 | 143 |
| Weighing (therapist or GP) before | 40.2 | 68.1 | -4 |
| Weighing (therapist or GP) during | 24.1 | 46.8 | -4 |
| Online add-on before | 4.5 | 0 | 22 |
| Online add-on during | 8.0 | 4.3 | 27 |

Notes: Percentages represent the combined endorsement rates of the two categories of agree/strongly agree, moderately worried/extremely worried, somewhat worsened/significantly worsened, and more/much more respectively. GP = general practitioner. NNT = number needed to take.

Supplemental Table 2: Mean helpfulness ratings as a function of age

|  | Adults  *M (SD)*  *n* | Adolescents  *M (SD)*  *n* | Cohen’s *d* |
| --- | --- | --- | --- |
| Strategies for interrupting thoughts and pathological behavior | 2.40 (0.94)  77 | 2.61 (1.03)  28 | 0.22 |
| Positive Thinking | 2.62 (1.10)  99 | 2.74 (1.22)  38 | 0.11 |
| Day planning | 3.39 (1.10)  103 | 3.45 (1.03)  38 | 0.06 |
| Daily routines | 3.45 (1.10)  106 | 3.50 (1.26)  40 | 0.04 |
| Enjoyable activities | 3.29 (1.26)  105 | 3.38 (1.17)  40 | 0.07 |
| Virtual social contact (friends) | 3.12 (1.19)  92 | 3.09 (1.31)  35 | -0.03 |
| Virtual social contact (family) | 3.04 (1.22)  71 | 2.67 (1.37)  24 | -0.29 |
| Mild physical exercises | 3.37 (1.25)  82 | 3.27 (1.17)  37 | -0.08 |
| Relaxation exercises | 2.84 (1.45)  57 | 2.87 (1.28)  30 | 0.02 |
| Meal planning | 2.97 (1.35)  93 | 3.16 (1.21)  31 | 0.14 |
| Yoga | 3.60 (1.34)  50 | 3.59 (1.18)  17 | -0.01 |
| Cooking with the family | 2.67 (1.30)  57 | 2.43 (1.40)  28 | -0.18 |
| Diaphragmatic and deep breathing | 2.74 (1.27)  43 | 2.23 (1.17)  13 | -0.41 |
| Meditation | 2.80 (1.45)  35 | 3.18 (1.25)  11 | 0.27 |
| Mindfulness | 2.68 (1.17)  59 | 3.33 (1.30)  12 | 0.55 |
| Seeking reinforcement and self-reinforcement | 2.51 (1.11)  70 | 2.79 (1.18)  24 | 0.25 |
| Keeping a diary of positive situations | 2.90 (1.19)  39 | 2.50 (1.77)  8 | -0.31 |
| Relaxing time with the family | 3.24 (1.15)  75 | 3.43 (1.03)  28 | 0.17 |
| Playing with the family | 3.22 (1.19)  46 | 2.91 (1.24)  23 | -0.26 |
| Opportunity to learn what you never had time to do | 2.81 (1.25)  62 | 3.46 (1.10)  26 | 0.54 |
| Pleasant activities on the internet | 2.71 (1.12)  58 | 2.95 (1.12)  21 | 0.21 |

Notes: Higher values represent higher perceived helpfulness. N’s are given for numbers of patients that used a specific strategy.

**2) Responses as a function of body weight**

Supplemental Table 3: Impact of the COVID-19 pandemic on patients with anorexia nervosa as a function of body weight

|  | BMI < 18.5  % | BMI > 18.5  % | NNT |
| --- | --- | --- | --- |
| **Overall impact** | | | |
| Worsening of eating disorder symptomatology | 46.7 | 37.7 | 11 |
| New symptoms | 17.4 | 24.6 | -14 |
| Worsening of quality of life | 56.5 | 45.9 | 9 |
| Impairment of therapy | 28.3 | 27.9 | 250 |
| **Eating disorder symptoms, exercise, and other eating-related behaviors** | | | |
| Eating disorder cognitions | | | |
| Drive for thinness | 63.0 | 67.2 | -24 |
| Fear of gaining weight | 68.5 | 73.8 | -19 |
| Body dissatisfaction | 67.4 | 65.6 | 56 |
| Eating concerns | 79.3 | 68.9 | 10 |
| Shape concerns | 75.0 | 68.9 | 16 |
| Weight concerns | 70.7 | 62.3 | 12 |
| Drive for physical activity | 77.2 | 72.1 | 20 |
| ED gives me control | 59.8 | 52.5 | 14 |
| ED gives me safety | 62.0 | 59.0 | 33 |
| Eating disorder behaviors | | | |
| Restrictive eating | 46.7 | 45.9 | 125 |
| Binge-eating (>1000 kcal) | 9.8 | 21.3 | -9 |
| Self-induced vomiting | 6.5 | 21.3 | -7 |
| Laxative abuse | 4.3 | 11.5 | -14 |
| Diuretic abuse | 3.3 | 1.6 | 59 |
| Weighing oneself | 37.0 | 21.3 | 6 |
| Hoarding food | 23.9 | 24.6 | -143 |
| Snacking/Unplanned eating | 16.3 | 36.1 | -5 |
| Skip meals | 34.8 | 36.1 | -77 |
| Sleep in and skip breakfast | 20.7 | 27.9 | -14 |
| Consuming triggering social media | 39.1 | 42.6 | -29 |
| Exercise behaviors | | | |
| Going for a walk | 76.1 | 63.9 | 8 |
| Jogging | 33.7 | 42.6 | -11 |
| Home-workouts | 63.0 | 65.6 | -38 |
| Standing on purpose | 37.0 | 19.7 | 6 |
| Taking stairs | 27.2 | 31.1 | -26 |
| Other eating-related behaviors | | | |
| Daily routine | 26.1 | 11.5 | 7 |
| Grocery shopping | 30.4 | 11.5 | 5 |
| Time for meal preparation | 51.1 | 44.3 | 15 |
| Going out for dinner | 4.3 | 0 | 23 |
| Cooking | 51.1 | 45.9 | 19 |
| Regular meal structure | 28.3 | 26.2 | 48 |
| Eating alone | 34.8 | 37.7 | -34 |
| **Weight change** | | | |
| Weight decrease | 23.9 | 11.5 | 8 |
| Weight maintenance | 71.7 | 78.7 | -14 |
| Weight increase | 4.3 | 9.8 | -18 |
| **General psychopathology symptoms** | | | |
| Depressive symptoms | | | |
| Sadness | 72.8 | 72.1 | 143 |
| Loss of pleasure | 68.5 | 62.3 | 16 |
| Loss of interest | 56.5 | 50.8 | 18 |
| Loss of energy | 63.0 | 54.1 | 11 |
| Loneliness | 77.2 | 77.0 | 500 |
| Sleep disturbances | 60.9 | 55.7 | 19 |
| Hypersomnia | 43.5 | 44.3 | -125 |
| Changes in appetite | 44.6 | 39.3 | 19 |
| Worthlessness | 56.5 | 57.4 | -111 |
| Suicidal thoughts | 33.7 | 37.7 | -25 |
| Anxieties | | | |
| Fear something bad may happen | 37.0 | 41.0 | -25 |
| Fear not being able to stop or control worries | 58.7 | 45.9 | 8 |
| Fear of contact with others | 39.1 | 36.1 | 33 |
| Worries that feelings get out of control | 51.1 | 52.5 | -71 |
| Other general psychopathology symptoms | | | |
| Motor restlessness | 66.3 | 55.7 | 9 |
| Inner restlessness | 80.4 | 60.7 | 5 |
| Loss of control | 63.0 | 63.9 | -111 |
| Concentration difficulty | 53.3 | 44.3 | 11 |
| Self-harm | 17.4 | 27.9 | -10 |
| Alcohol | 9.8 | 9.8 |  |
| **Worries** | | | |
| Own infection | 19.6 | 16.4 | 31 |
| Infection of others (e.g. family or friends) | 52.2 | 52.2 |  |
| To infect others | 39.1 | 41 | -53 |
| Negative impact on therapy | 27.2 | 29.5 | -43 |
| Relapse | 52.2 | 49.2 | 33 |
| Food insecurity (i.e. availability, access) | 16.3 | 23.0 | -15 |
| Financial situation | 17.4 | 24.6 | -14 |
| Loss of job | 8.7 | 13.1 | -23 |
| **Interpersonal conflicts** | | | |
| Relationship conflicts | 15.2 | 8.2 | 14 |
| Friendship conflicts | 13.0 | 14.8 | -56 |
| Family conflicts | 52.2 | 39.3 | 8 |
| Conflicts in the workplace | 6.5 | 3.3 | 31 |
| **Health care utilization before and during the COVID-19 pandemic** | | | |
| In-person psychotherapy before | 92.4 | 80.3 | 8 |
| In-person psychotherapy during | 60.9 | 49.2 | 9 |
| Videoconference therapy before | 0 | 3.3 | -30 |
| Videoconference therapy during | 23.9 | 31.1 | -14 |
| Telephone contact before | 10.9 | 13.1 | -45 |
| Telephone contact during | 42.4 | 23.0 | 5 |
| GP visit before | 47.8 | 39.3 | 12 |
| GP visit during | 29.3 | 16.4 | 8 |
| Weighing (therapist or GP) before | 59.8 | 32.8 | 4 |
| Weighing (therapist or GP) during | 37.0 | 21.3 | 6 |
| Add-on online intervention before | 3.3 | 3.3 |  |
| Add-on online intervention during | 9.8 | 3.3 | 15 |

Notes: Percentages represent the combined endorsement rates of the two categories of agree/strongly agree, moderately worried/extremely worried, somewhat worsened/significantly worsened, and more/much more respectively. GP = general practitioner. NNT = number needed to take.

Supplemental Table 4: Mean helpfulness ratings as a function of body weight

|  | BMI < 18.5  *M (SD)*  *n* | BMI > 18.5  *M (SD)*  *n* | Cohen’s *d* |
| --- | --- | --- | --- |
| Strategies for interrupting thoughts and pathological behavior | 2.44 (0.91)  63 | 2.48 (1.06)  40 | 0.04 |
| Positive Thinking | 2.58 (1.10)  82 | 2.69 (1.16)  49 | 0.10 |
| Day planning | 3.39 (1.02)  82 | 3.40 (1.18)  53 | 0.01 |
| Daily routines | 3.37 (1.16)  89 | 3.57 (1.14)  51 | 0.17 |
| Enjoyable activities | 3.26 (1.27)  85 | 3.35 (1.17)  54 | 0.07 |
| Virtual social contact (friends) | 3.17 (1.13)  75 | 2.96 (1.35)  48 | -0.17 |
| Virtual social contact (family) | 2.94 (1.19)  63 | 2.79 (1.37)  29 | -0.12 |
| Mild physical exercises | 3.40 (1.21)  70 | 3.18 (1.23)  45 | -0.18 |
| Relaxation exercises | 3.22 (1.29)  51 | 2.39 (1.41)  33 | -0.62 |
| Meal planning | 2.88 (1.28)  78 | 3.32 (1.29)  41 | 0.34 |
| Yoga | 3.80 (1.36)  40 | 3.30 (1.14)  27 | -0.39 |
| Cooking with the family | 2.61 (1.27)  51 | 2.52 (1.43)  31 | -0.01 |
| Diaphragmatic and deep breathing | 2.79 (1.39)  33 | 2.39 (1.03)  23 | -0.32 |
| Meditation | 3.07 (1.36)  30 | 2.40 (1.35)  15 | -0.49 |
| Mindfulness | 2.98 (1.25)  41 | 2.48 (1.05)  27 | -0.43 |
| Seeking reinforcement and self-reinforcement | 2.59 (1.14)  58 | 2.53 (1.08)  34 | -0.05 |
| Keeping a diary of positive situations | 2.89 (1.45)  27 | 2.74 (1.10)  19 | -0.11 |
| Relaxing time with the family | 3.29 (1.11)  59 | 3.32 (1.11)  41 | 0.03 |
| Playing with the family | 2.98 (1.18)  42 | 3.33 (1.24)  24 | 0.29 |
| Opportunity to learn what you never had time to do | 2.98 (1.20)  46 | 2.98 (1.27)  41 | 0.00 |
| Pleasant activities on the internet | 2.79 (1.08)  47 | 2.67 (1.15)  30 | -0.11 |

Notes: Higher values represent higher perceived helpfulness. N’s are given for numbers of patients that used a specific strategy.
